# Supplementary material for: Novel 2-alkythio-4-chloro-N-[imino(heteroaryl)methyl]benzenesulfonamide Derivatives: Synthesis, Molecular Structure, Anticancer Activity and Metabolic Stability
Source: Int J Mol Sci. 2023 Jun 5;24(11):9768. doi: 10.3390/ijms24119768 (PMC10253812; doi:10.3390/ijms24119768)
Supplement: Supplementary file 1 [file ijms-24-09768-s001.zip › ijms-2415797-supplementary.pdf]

Supplementary material

Novel 2-alkythio-4-chloro-*N*-[imino(heteroaryl)methyl]benzenesulfonamide derivatives: synthesis, molecular structure, anticancer activity and metabolic stability

Beata Żołnowska [1,*], Jarosław Sławiński [1], Mariusz Belka [2], Tomasz Bączek [2], Jarosław Chojnacki [3] and Anna Kawiak [4,*]

[1] Department of Organic Chemistry, Medical University of Gdańsk, Al. Gen. J. Hallera 107, 80-416 Gdańsk, Poland; beata.zolnowska@gumed.edu.pl, jaroslaw.slawinski@gumed.edu.pl
[2] Department of Pharmaceutical Chemistry, Medical University of Gdańsk, Al. Gen. J. Hallera 107, 80-416 Gdańsk, Poland; mariusz.belka@gumed.edu.pl, tbaczek@gumed.edu.pl
[3] Department of Inorganic Chemistry, Gdańsk University of Technology, ul. Narutowicza 11/12, 80-233 Gdańsk, Poland; jaroslaw.chojnacki@pg.edu.pl
[4] Department of Biotechnology, Intercollegiate Faculty of Biotechnology, University of Gdańsk and Medi-cal University of Gdańsk, ul. Abrahama 58, 80-307 Gdańsk, Poland; anna.kawiak@biotech.ug.edu.pl

* Correspondence: beata.zolnowska@gumed.edu.pl (BZ); anna.kawiak@biotech.ug.edu.pl (AK)

Contents:

Table S1. Crystal data and structure refinement details for **10**.

Table S2. Hydrogen-bond geometry (Å, °) for **10**.

^{1}H NMR of compd **9** (500 MHz, DMSO-*d*$_6$).

^{13}C NMR of compd **9** (125 MHz, DMSO-*d*$_6$).

^{1}H NMR of compd **11** (500 MHz, DMSO-*d*$_6$).

^{13}C NMR of compd **11** (150.9 MHz, DMSO-*d*$_6$).

^{1}H NMR of compd **12** (500 MHz, DMSO-*d*$_6$).

^{13}C NMR of compd **12** (150.9 MHz, DMSO-*d*$_6$).

^{1}H NMR of compd **13** (500 MHz, DMSO-*d*$_6$).

^{13}C NMR of compd **13** (125 MHz, DMSO-*d*$_6$).

^{1}H NMR of compd **14** (500 MHz, DMSO-*d*$_6$).

^{13}C NMR of compd **14** (150.9 MHz, DMSO-*d*$_6$).

^{1}H NMR of compd **15** (200 MHz, DMSO-*d*$_6$).

^{13}C NMR of compd **15** (125 MHz, DMSO-*d*$_6$).

^{1}H NMR of compd **21** (500 MHz, DMSO-*d*$_6$).

^{13}C NMR of compd **21** (125 MHz, DMSO-*d*$_6$).

^{1}H NMR of compd **22** (500 MHz, DMSO-*d*$_6$).

^{13}C NMR of compd **22** (125 MHz, DMSO-*d*$_6$).

^{1}H NMR of compd **23** (500 MHz, DMSO-*d*$_6$).

^{13}C NMR of compd **23** (125 MHz, DMSO-*d*$_6$).

Table S1. Crystal data and structure refinement details for **10**.

Crystal data	
Chemical formula	$C_{20}H_{18}ClF_3N_4O_2S_3$
M_r	535.01
Crystal system, space group	Monoclinic, $P2_1/c$
Temperature (K)	120
a, *b*, *c* (Å)	10.215 (2), 24.427 (3), 10.310 (2)
β (°)	116.449 (15)
V (Å^3)	2303.3 (8)
Z	4
Radiation type	Cu *K*α
μ (mm^{-1})	4.47
Crystal size (mm)	0.34 × 0.13 × 0.11
Data collection	
Diffractometer	STOE *IPDS* 2T
Absorption correction	Integration<br>STOE *X-RED32*, absorption correction by Gaussian integration, analogous to P. Coppens, "The Evaluation of Absorption and Extinction in Single-Crystal Structure Analysis", published in F. R. Ahmed (Editor), "Crystallographic Computing", Munksgaard, Copenhagen (1970), 255 – 270
T_{min}, T_{max}	0.223, 0.748
No. of measured, independent and observed [$I > 2\sigma(I)$] reflections	11762, 3416, 3089
R_{int}	0.028

θ_{max} (°)	60.0
$(\sin \theta/\lambda)_{max}$ (Å^{-1})	0.562
Refinement	
$R[F^2 > 2\sigma(F^2)]$, $wR(F^2)$, S	0.034, 0.098, 1.07
No. of reflections	3416
No. of parameters	336
No. of restraints	44
H-atom treatment	H atoms treated by a mixture of independent and constrained refinement
$\Delta\rangle_{max}$, $\Delta\rangle_{min}$ (e Å^{-3})	0.30, -0.47

Table S2. Hydrogen-bond geometry (Å, º) for **10**.

D—H···*A*	*D*—H	H···*A*	*D*···*A*	*D*—H···*A*
N2—H2*A*···O1	0.89 (2)	2.09 (2)	2.785 (2)	134 (2)
N2—H2*B*···S3	0.86 (2)	2.28 (2)	3.034 (2)	147 (2)

Computer programs: *X-AREA* WinXpose 2.0.22.0 (STOE, 2016), *X-AREA* Recipe 1.33.0.0 (STOE, 2015), STOE *X-AREA*, ShelXT [40], *SHELXL* [41], Olex2 [42].

40. Sheldrick, G.M. SHELXT – Integrated space-group and crystal-structure determination. *Acta Cryst.* **2015**, *A71*, 3–8.
41. Sheldrick, G.M. Crystal structure refinement with SHELXL. *Acta Cryst.* **2015**, *C71*, 3–8.
42. Dolomanov, O.V.; Bourhis, L.J.; Gildea, R.J.; Howard, J.A.K.; Puschmann, H. OLEX2: a complete structure solution, refinement and analysis program. *J. Appl. Cryst.* **2009**, *42*, 339–341.

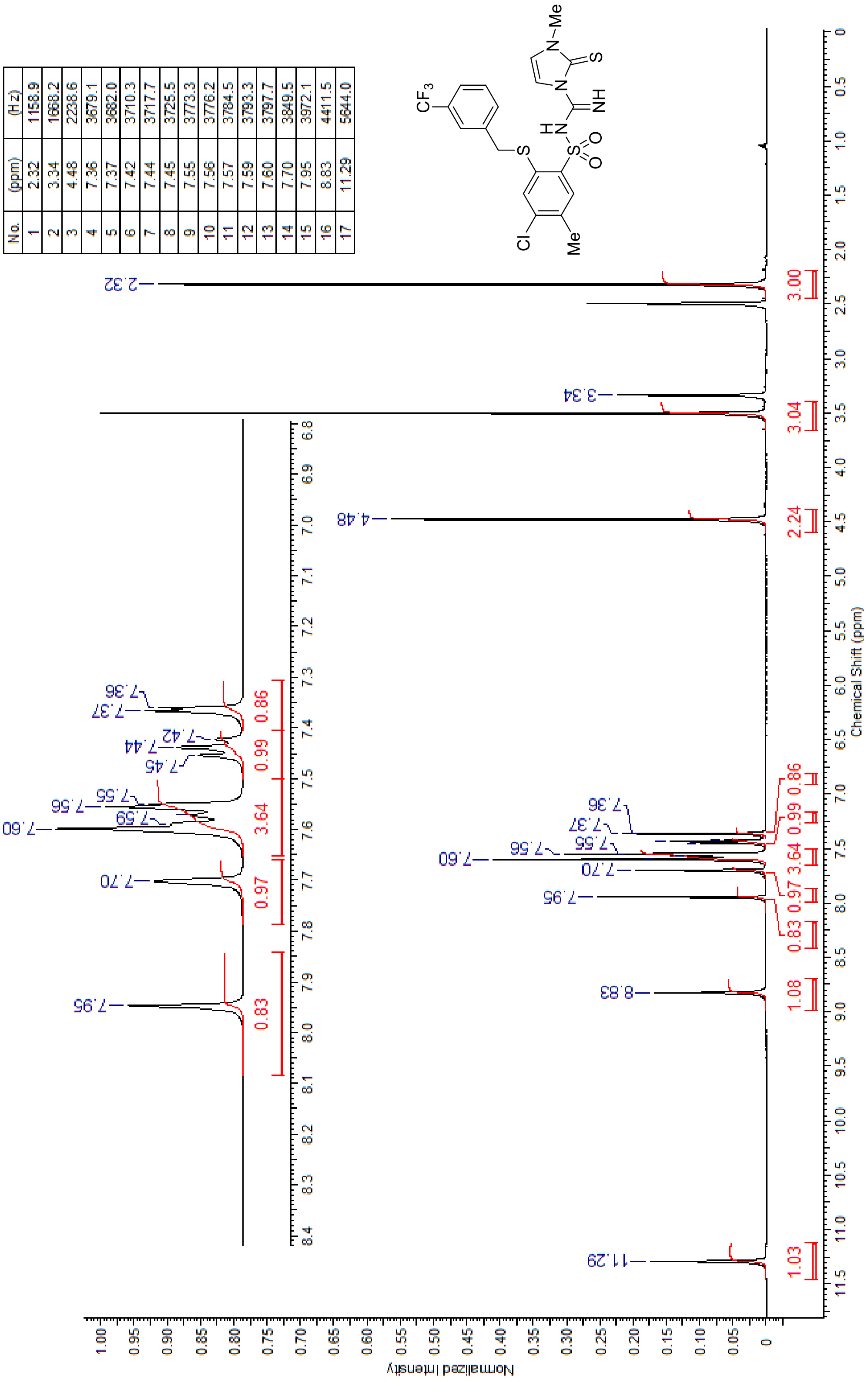

No.	(ppm)	(Hz)
1	2.32	1158.9
2	3.34	1668.2
3	4.48	2238.6
4	7.36	3679.1
5	7.37	3682.0
6	7.42	3710.3
7	7.44	3717.7
8	7.45	3725.5
9	7.55	3773.3
10	7.56	3776.2
11	7.57	3784.5
12	7.59	3793.3
13	7.60	3797.7
14	7.70	3849.5
15	7.95	3972.1
16	8.83	4411.5
17	11.29	5644.0

^{1}H NMR of compd **9** (500 MHz, DMSO-d_6).

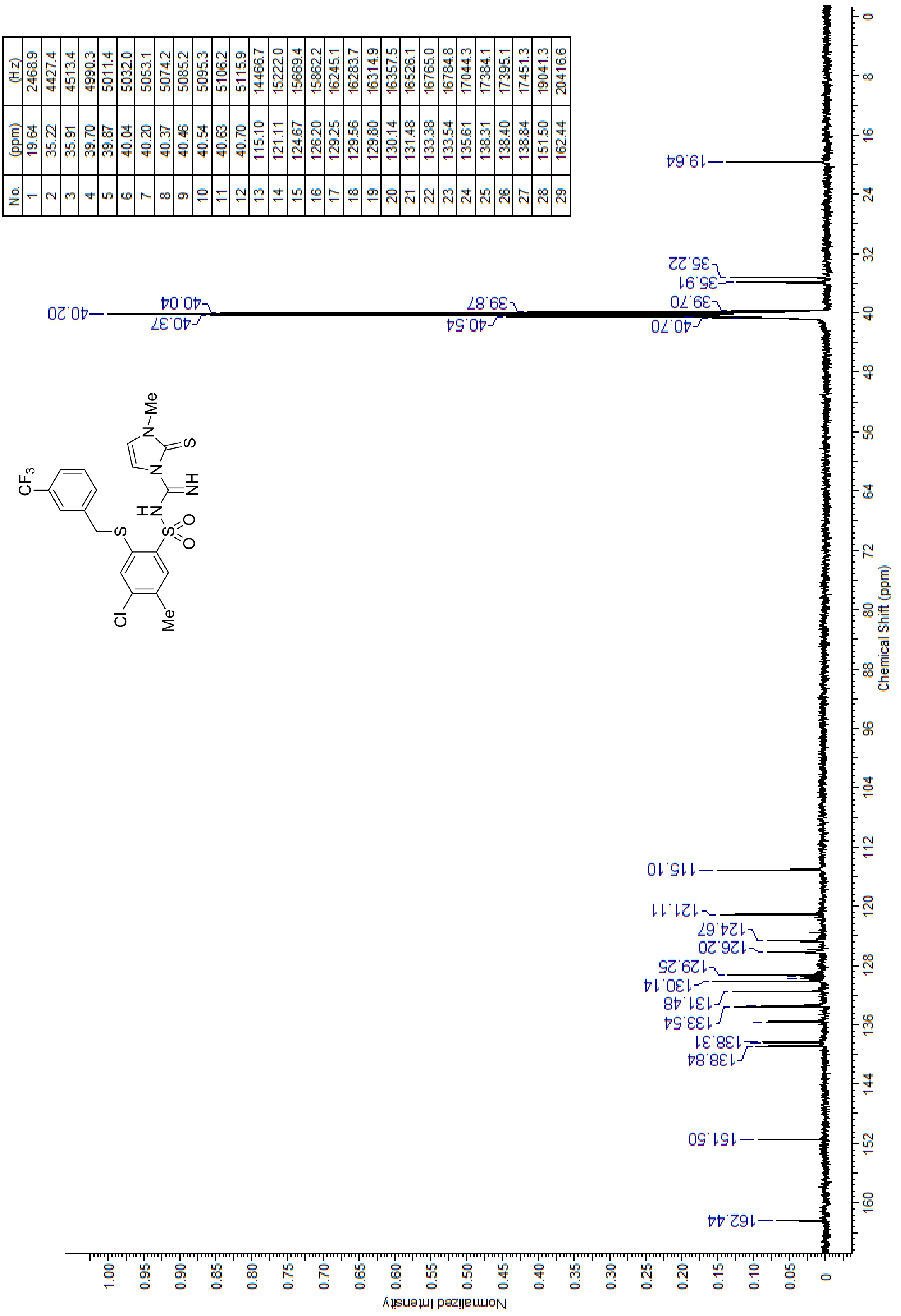

No.	(ppm)	(Hz)
1	19.64	2468.9
2	35.22	4427.4
3	35.91	4513.4
4	39.70	4990.3
5	39.87	5011.4
6	40.04	5032.0
7	40.20	5053.1
8	40.37	5074.2
9	40.46	5085.2
10	40.54	5095.3
11	40.63	5106.2
12	40.70	5115.9
13	115.10	14466.7
14	121.11	15222.0
15	124.67	15669.4
16	126.20	15862.2
17	129.25	16245.1
18	129.56	16283.7
19	129.80	16314.9
20	130.14	16357.5
21	131.48	16526.1
22	133.38	16765.0
23	133.54	16784.8
24	135.61	17044.3
25	138.31	17384.1
26	138.40	17395.1
27	138.84	17451.3
28	151.50	19041.3
29	162.44	20416.6

^{13}C NMR of compd **9** (125 MHz, DMSO-d_6).

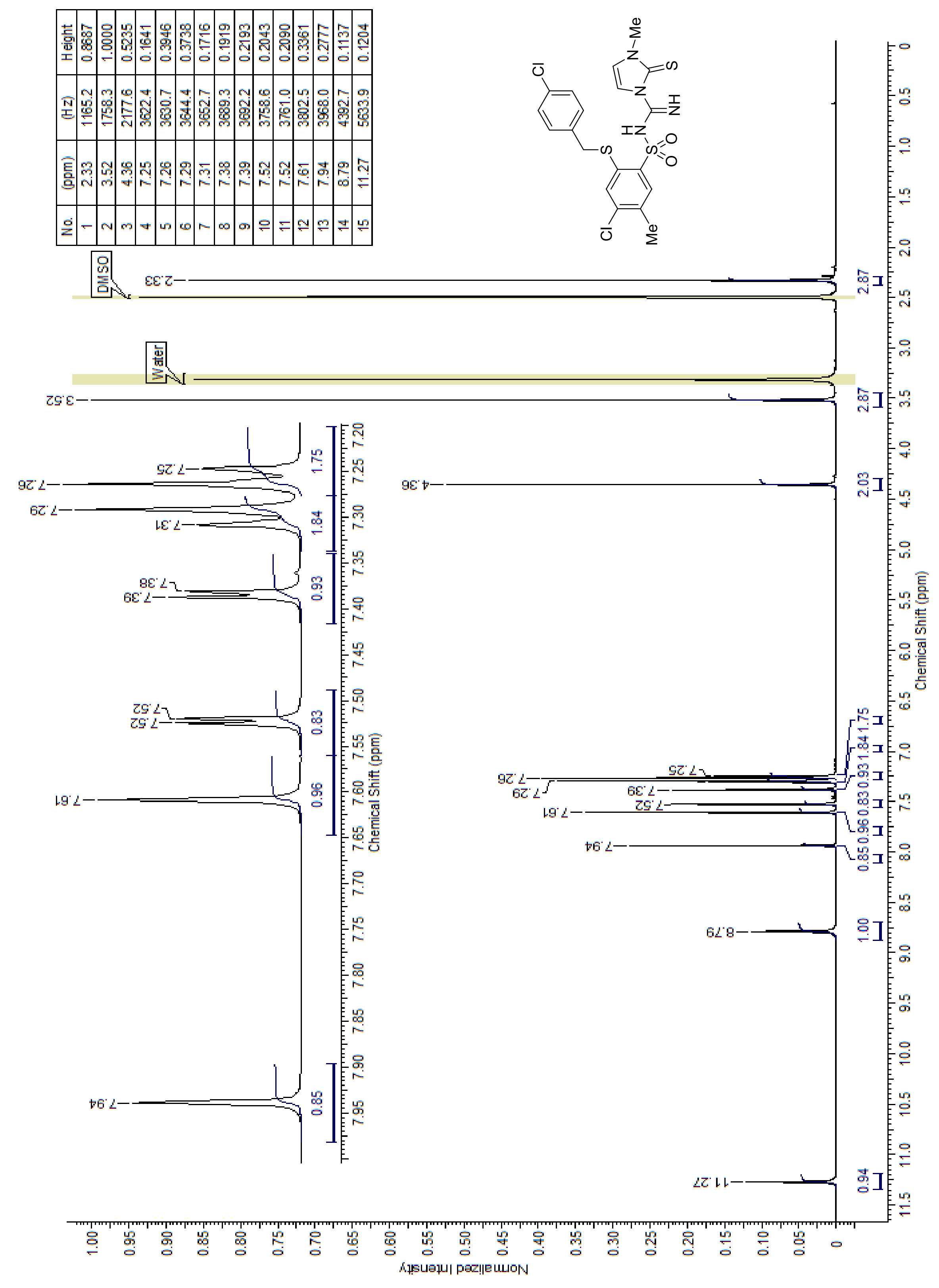

^{1}H NMR of compd **11** (500 MHz, DMSO-d_6).

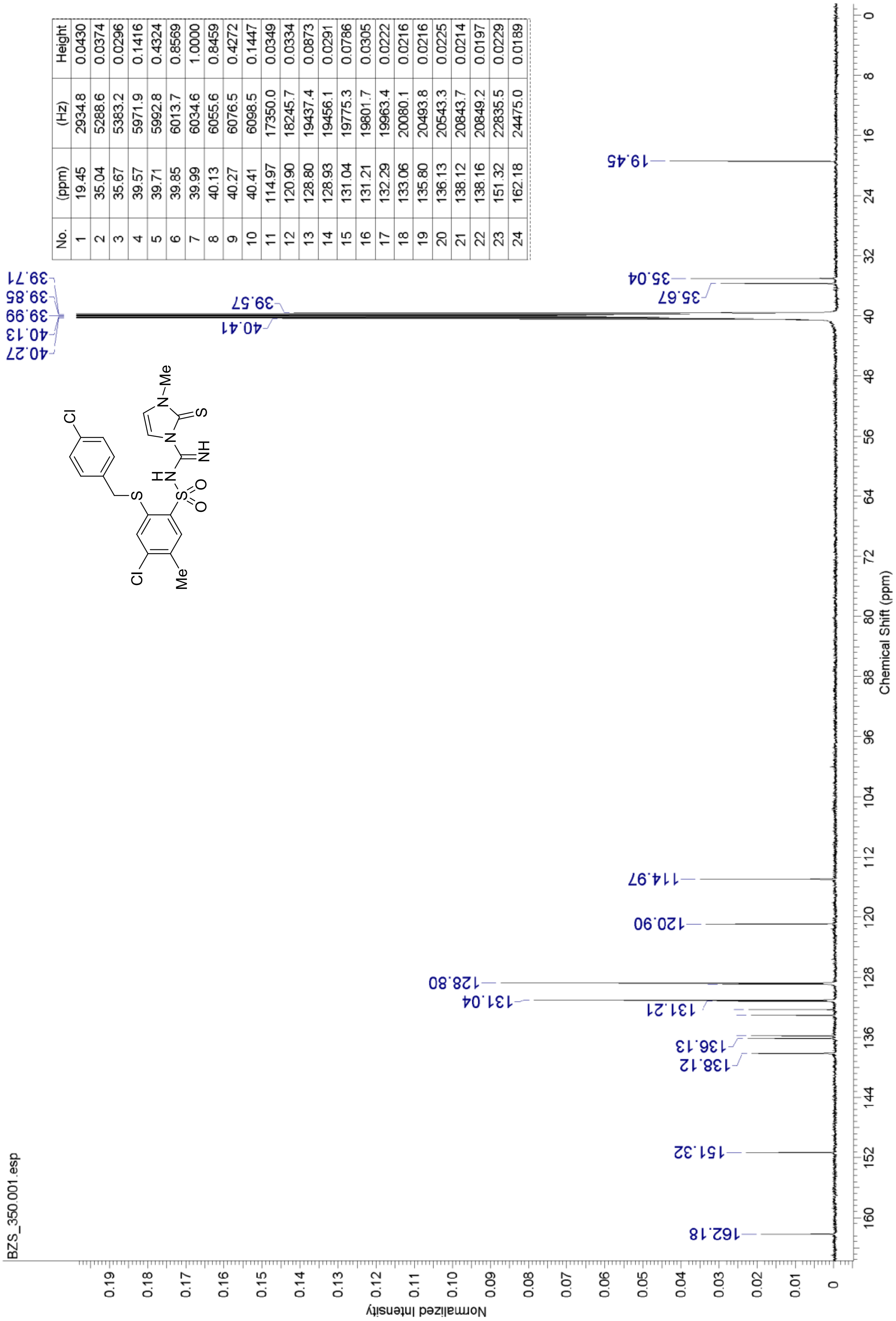

No.	(ppm)	(Hz)	Height
1	19.45	2934.8	0.0430
2	35.04	5288.6	0.0374
3	35.67	5383.2	0.0296
4	39.57	5971.9	0.1416
5	39.71	5992.8	0.4324
6	39.85	6013.7	0.8569
7	39.99	6034.6	1.0000
8	40.13	6055.6	0.8459
9	40.27	6076.5	0.4272
10	40.41	6098.5	0.1447
11	114.97	17350.0	0.0349
12	120.90	18245.7	0.0334
13	128.80	19437.4	0.0873
14	128.93	19456.1	0.0291
15	131.04	19775.3	0.0786
16	131.21	19801.7	0.0305
17	132.29	19963.4	0.0222
18	133.06	20080.1	0.0216
19	135.80	20493.8	0.0216
20	136.13	20543.3	0.0225
21	138.12	20843.7	0.0214
22	138.16	20849.2	0.0197
23	151.32	22835.5	0.0229
24	162.18	24475.0	0.0189

^{13}C NMR of compd **11** (150.9 MHz, DMSO-d_6).

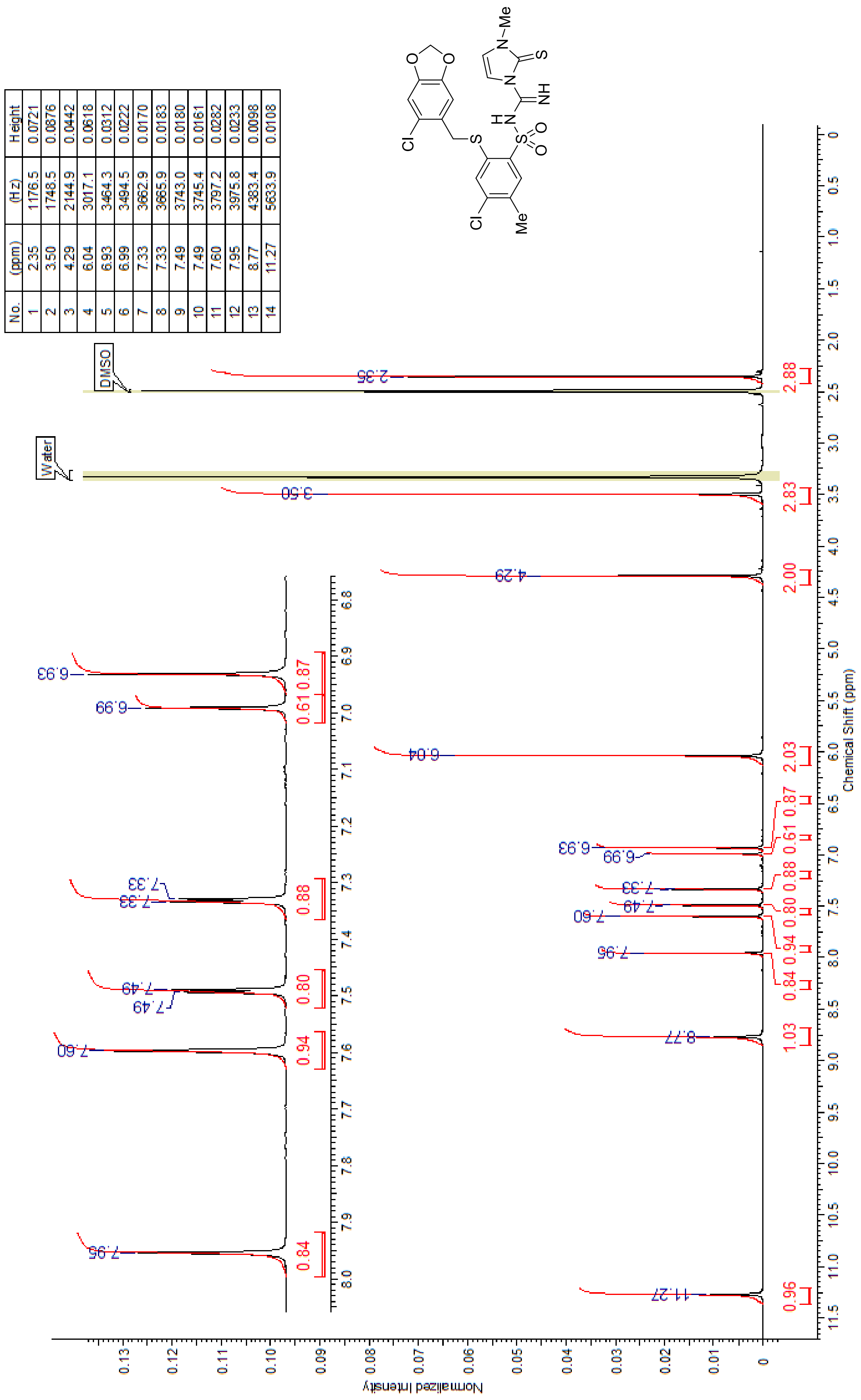

^{1}H NMR of compd **12** (500 MHz, DMSO-d_6).

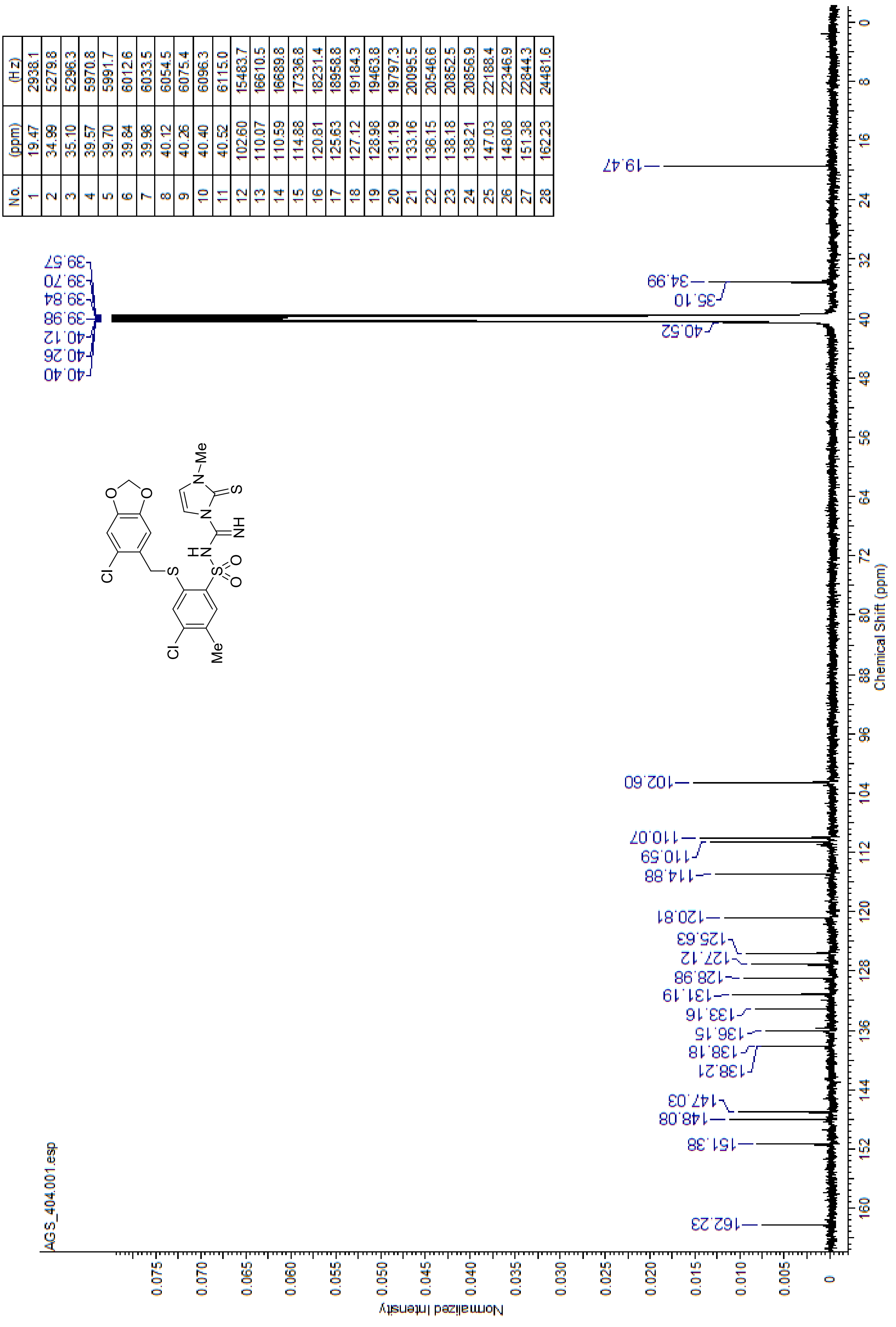

No.	(ppm)	(Hz)
1	19.47	2938.1
2	34.99	5279.8
3	35.10	5296.3
4	39.57	5970.8
5	39.70	5991.7
6	39.84	6012.6
7	39.98	6033.5
8	40.12	6054.5
9	40.26	6075.4
10	40.40	6096.3
11	40.52	6115.0
12	102.60	15483.7
13	110.07	16610.5
14	110.59	16689.8
15	114.88	17336.8
16	120.81	18231.4
17	125.63	18958.8
18	127.12	19184.3
19	128.98	19463.8
20	131.19	19797.3
21	133.16	20095.5
22	136.15	20546.6
23	138.18	20852.5
24	138.21	20856.9
25	147.03	22188.4
26	148.08	22346.9
27	151.38	22844.3
28	162.23	24481.6

^{13}C NMR of compd **12** (150.9 MHz, DMSO-d_6).

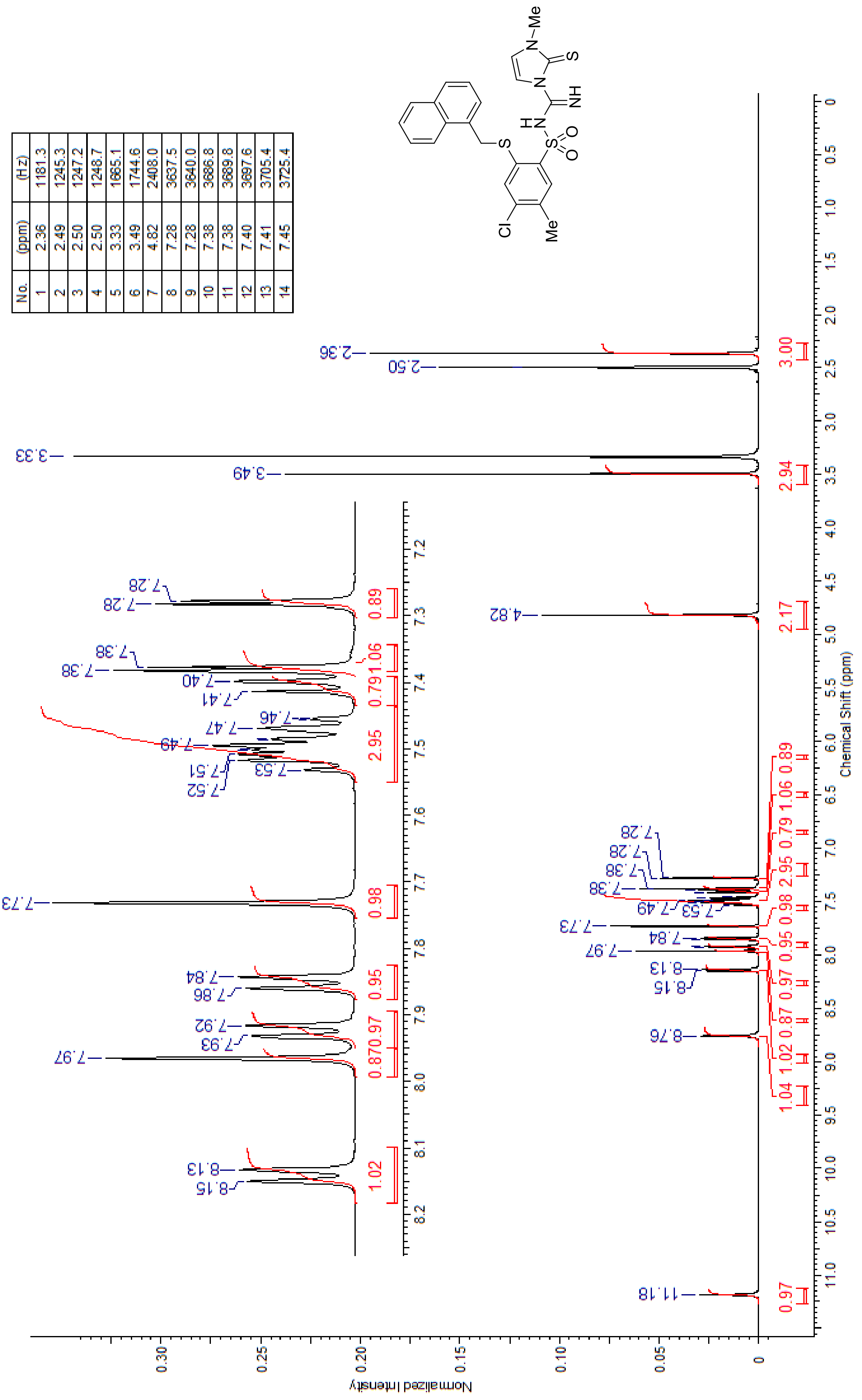

^{1}H NMR of compd **13** (500 MHz, DMSO-d_6).

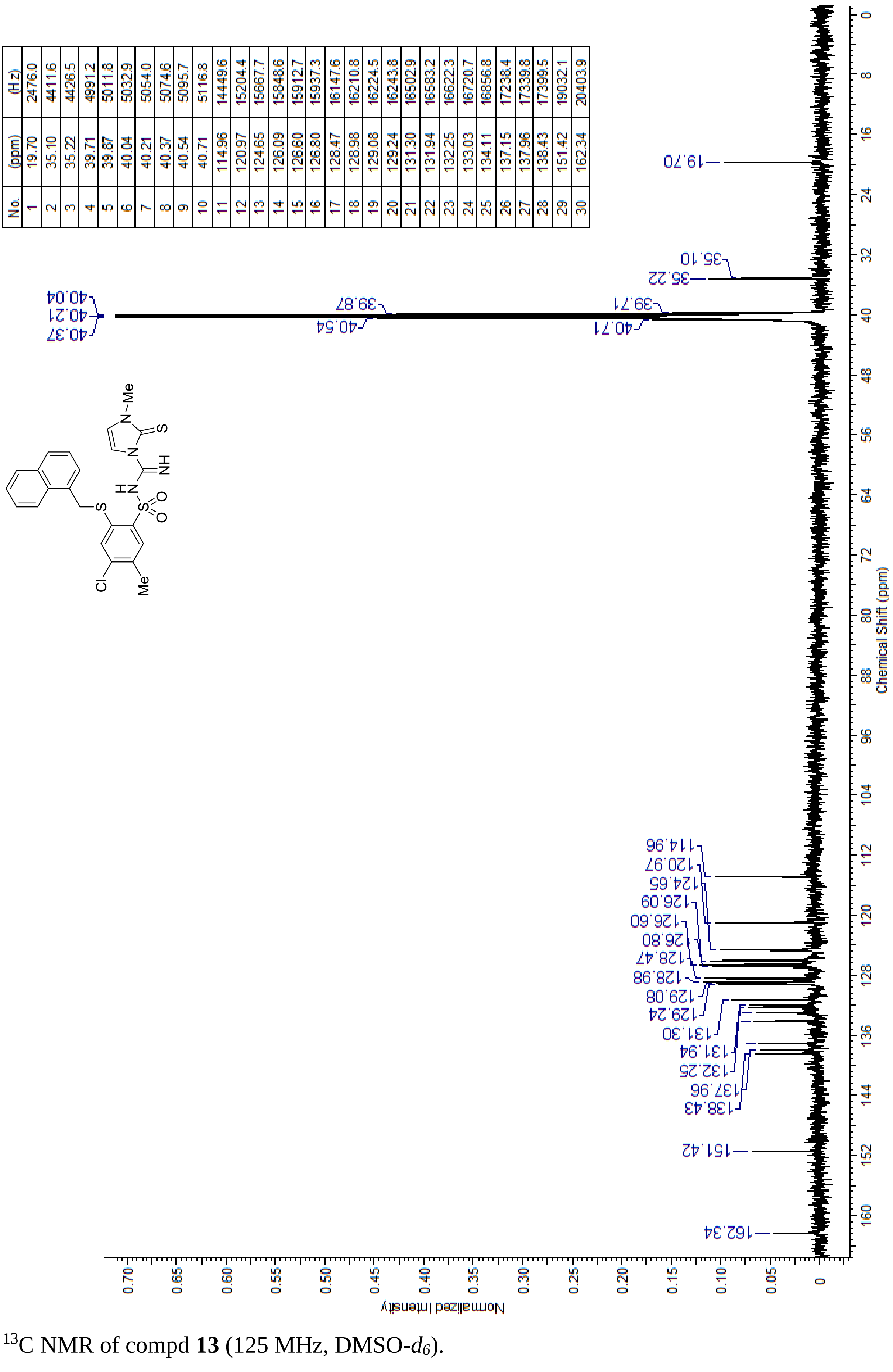

No.	(ppm)	(Hz)
1	19.70	2476.0
2	35.10	4411.6
3	35.22	4426.5
4	39.71	4991.2
5	39.87	5011.8
6	40.04	5032.9
7	40.21	5054.0
8	40.37	5074.6
9	40.54	5095.7
10	40.71	5116.8
11	114.96	14449.6
12	120.97	15204.4
13	124.65	15667.7
14	126.09	15848.6
15	126.60	15912.7
16	126.80	15937.3
17	128.47	16147.6
18	128.98	16210.8
19	129.08	16224.5
20	129.24	16243.8
21	131.30	16502.9
22	131.94	16583.2
23	132.25	16622.3
24	133.03	16720.7
25	134.11	16856.8
26	137.15	17238.4
27	137.96	17339.8
28	138.43	17399.5
29	151.42	19032.1
30	162.34	20403.9

^{13}C NMR of compd **13** (125 MHz, DMSO-d_6).

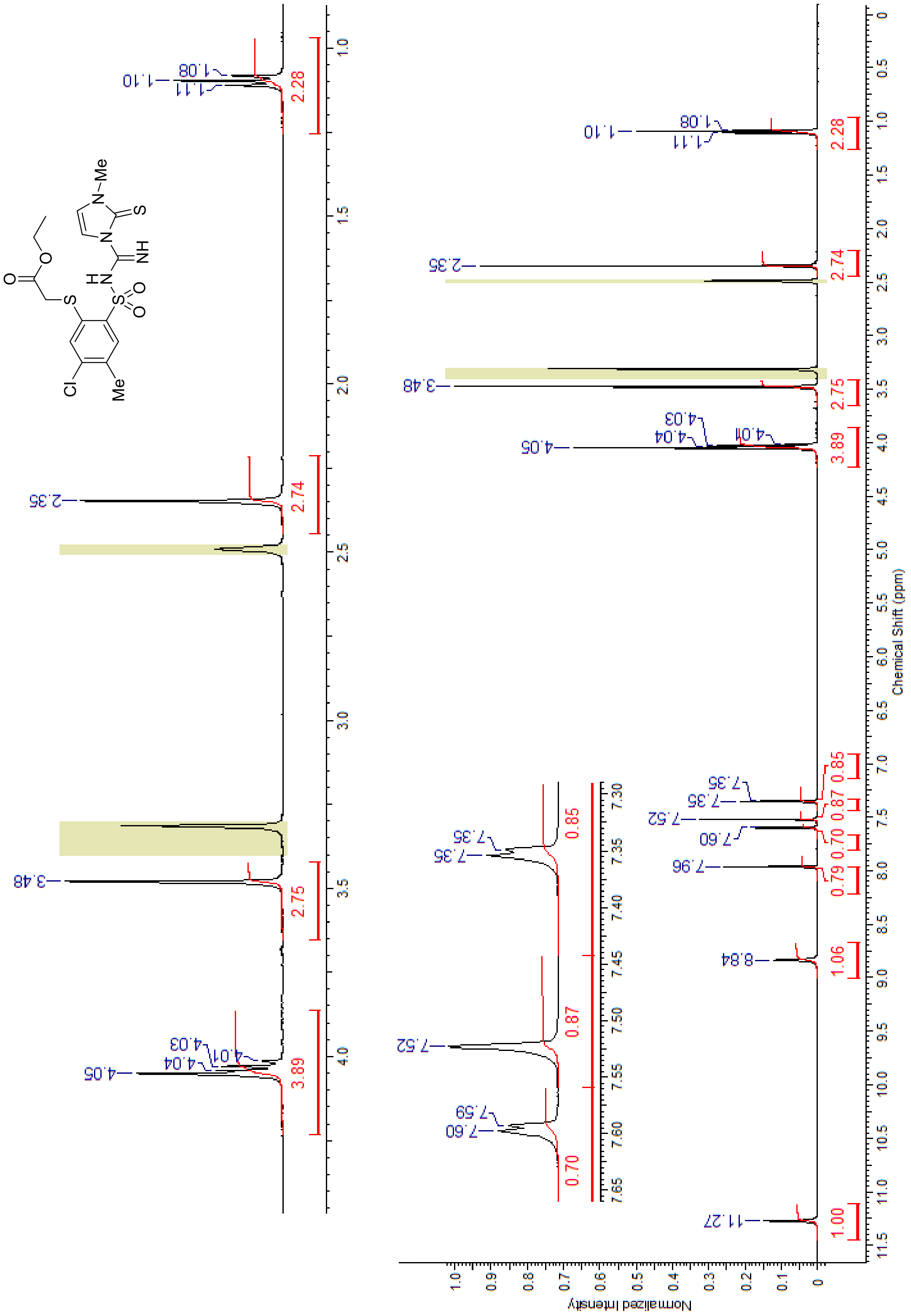

^{1}H NMR of compd **14** (500 MHz, DMSO-d_6).

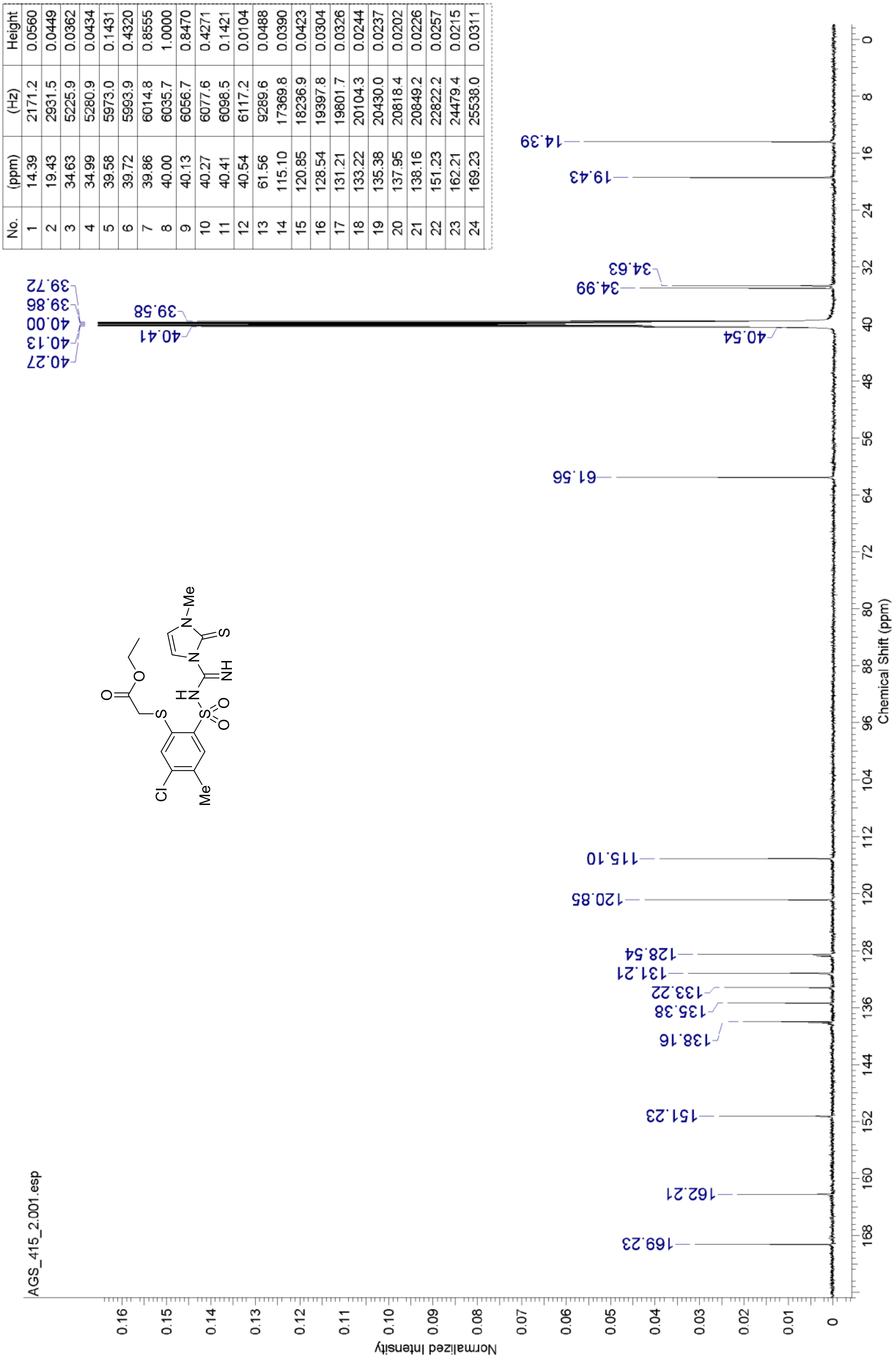

No.	(ppm)	(Hz)	Height
1	14.39	2171.2	0.0560
2	19.43	2931.5	0.0449
3	34.63	5225.9	0.0362
4	34.99	5280.9	0.0434
5	39.58	5973.0	0.1431
6	39.72	5993.9	0.4320
7	39.86	6014.8	0.8555
8	40.00	6035.7	1.0000
9	40.13	6056.7	0.8470
10	40.27	6077.6	0.4271
11	40.41	6098.5	0.1421
12	40.54	6117.2	0.0104
13	61.56	9289.6	0.0488
14	115.10	17369.8	0.0390
15	120.85	18236.9	0.0423
16	128.54	19397.8	0.0304
17	131.21	19801.7	0.0326
18	133.22	20104.3	0.0244
19	135.38	20430.0	0.0237
20	137.95	20818.4	0.0202
21	138.16	20849.2	0.0226
22	151.23	22822.2	0.0257
23	162.21	24479.4	0.0215
24	169.23	25538.0	0.0311

^{13}C NMR of compd **14** (150.9 MHz, DMSO-d_6).

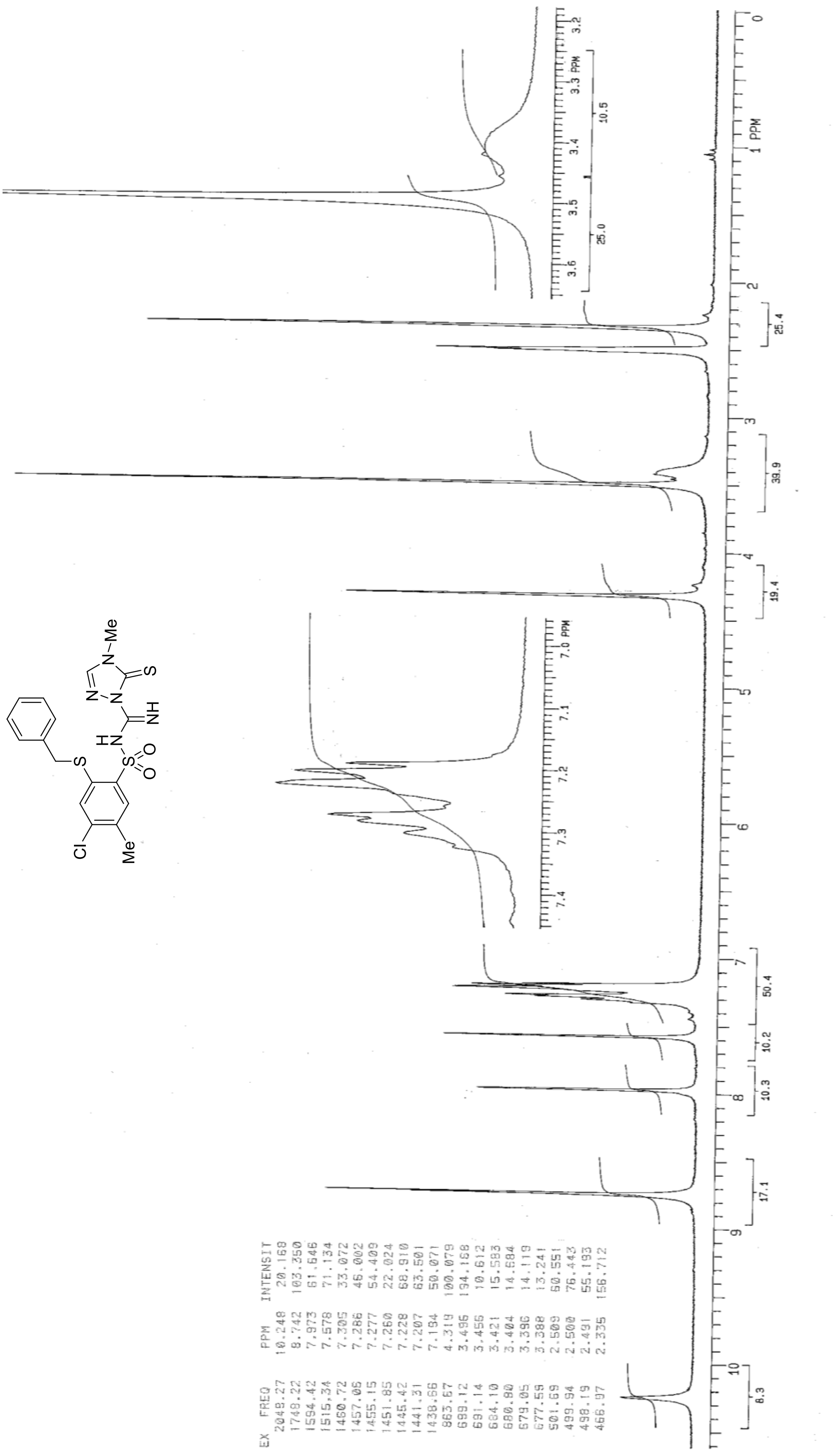

^{1}H NMR of compd **15** (50 MHz, DMSO-d_6).

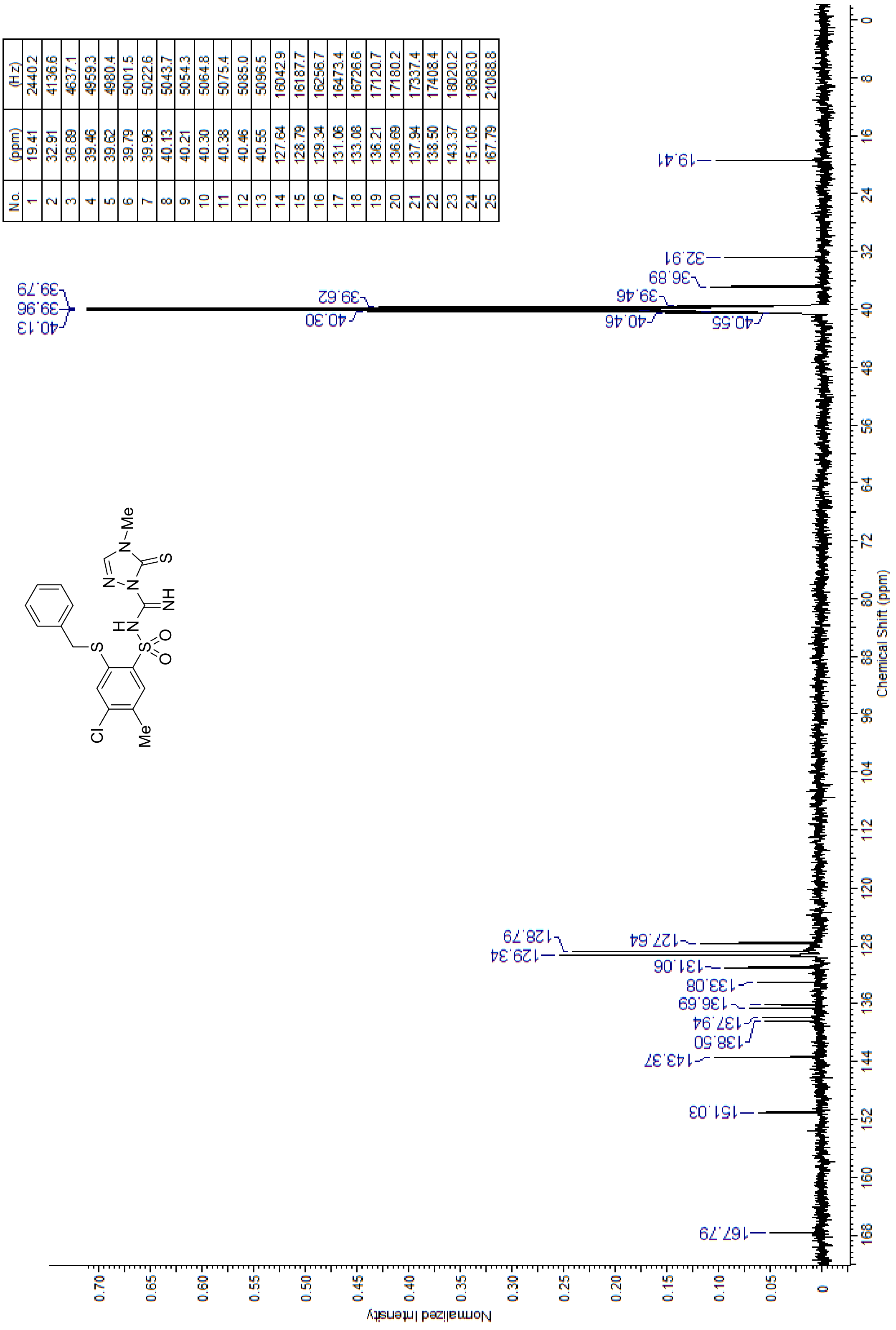

^{13}C NMR of compd **15** (125 MHz, DMSO-d_6).

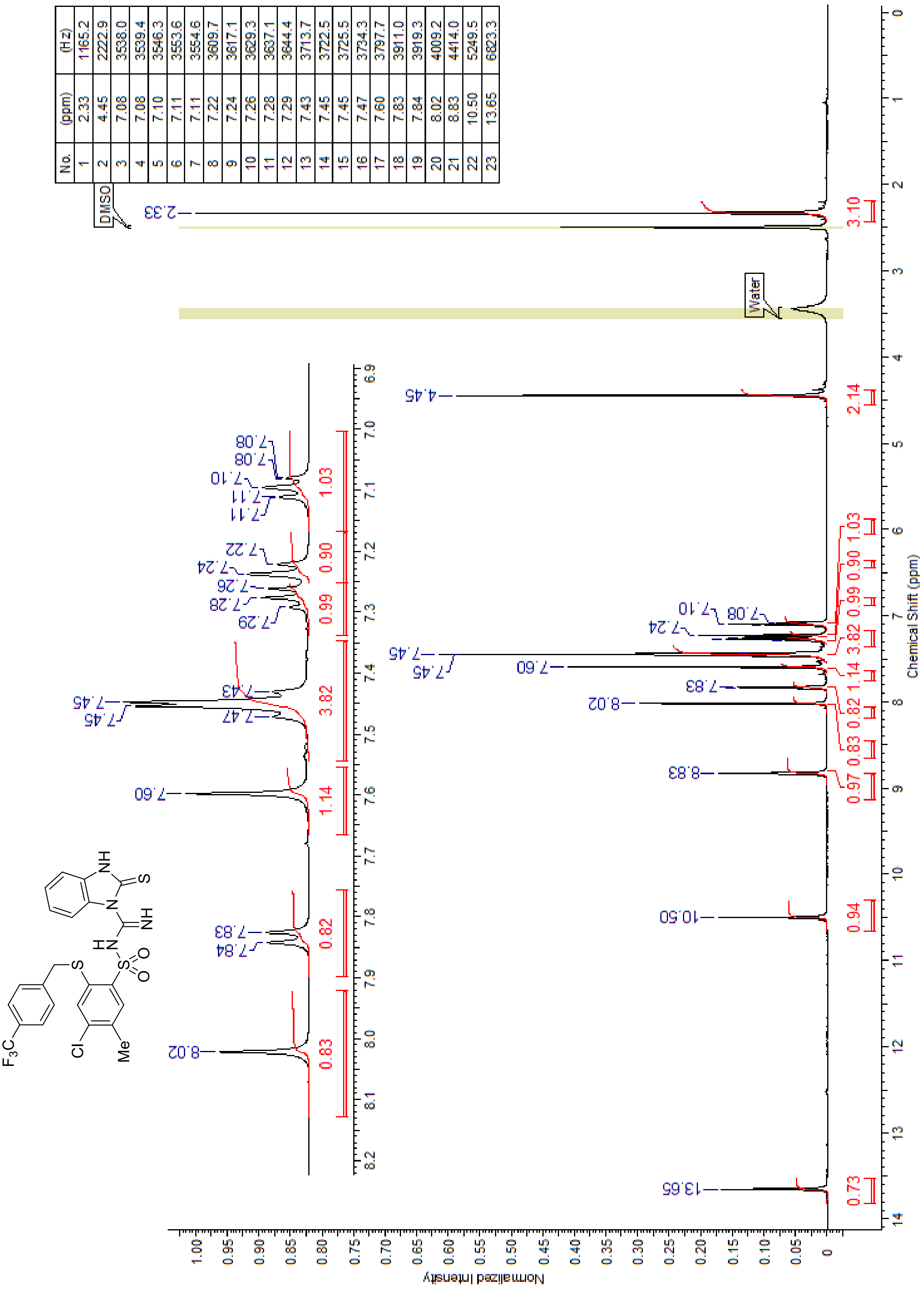

No.	(ppm)	(Hz)
1	2.33	1165.2
2	4.45	2222.9
3	7.08	3538.0
4	7.08	3539.4
5	7.10	3546.3
6	7.11	3553.6
7	7.11	3554.6
8	7.22	3609.7
9	7.24	3617.1
10	7.26	3629.3
11	7.28	3637.1
12	7.29	3644.4
13	7.43	3713.7
14	7.45	3722.5
15	7.45	3725.5
16	7.47	3734.3
17	7.60	3797.7
18	7.83	3911.0
19	7.84	3919.3
20	8.02	4009.2
21	8.83	4414.0
22	10.50	5249.5
23	13.65	6823.3

^{1}H NMR of compd **21** (500 MHz, DMSO-d_6).

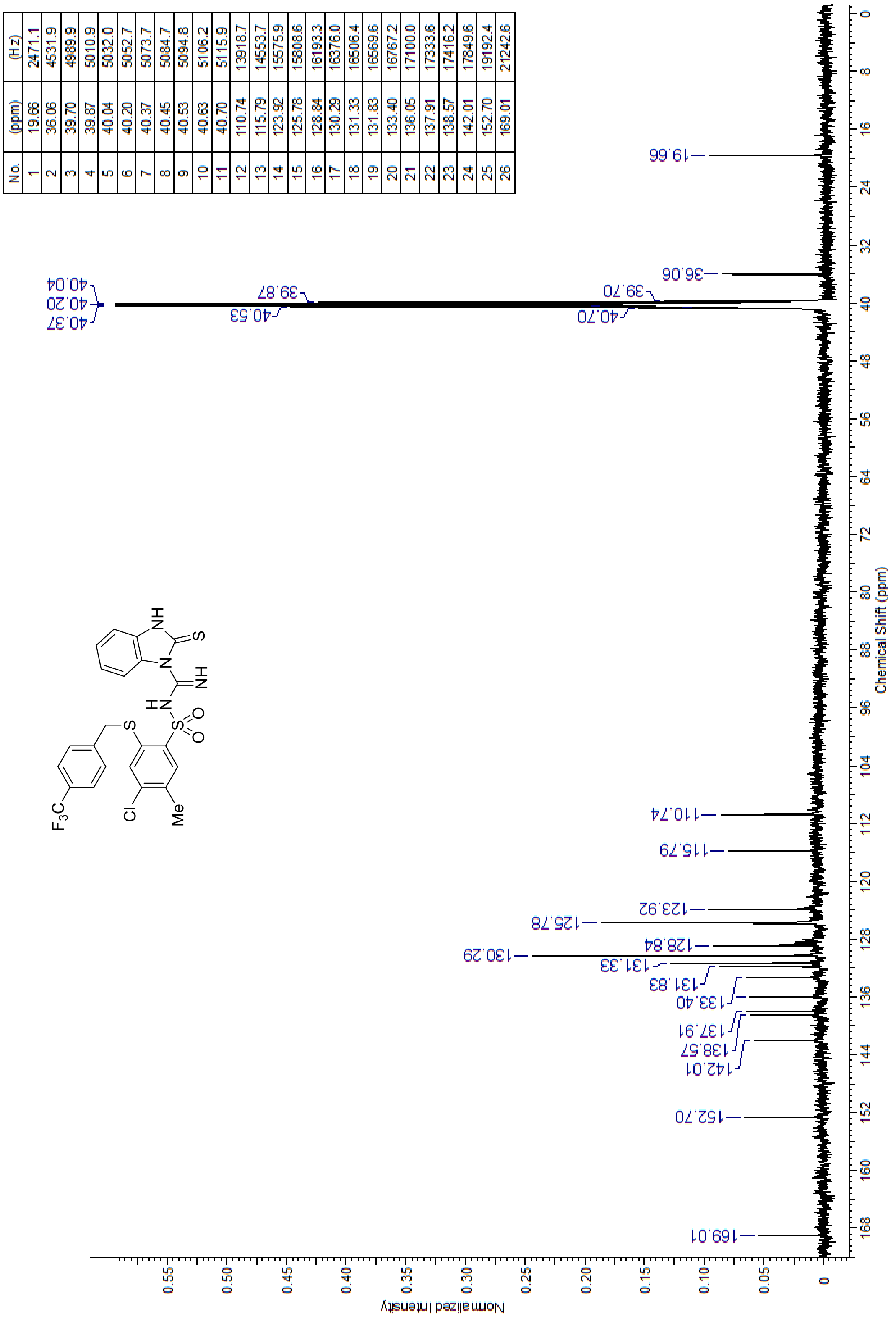

No.	(ppm)	(Hz)
1	19.66	2471.1
2	36.06	4531.9
3	39.70	4989.9
4	39.87	5010.9
5	40.04	5032.0
6	40.20	5052.7
7	40.37	5073.7
8	40.45	5084.7
9	40.53	5094.8
10	40.63	5106.2
11	40.70	5115.9
12	110.74	13918.7
13	115.79	14553.7
14	123.92	15575.9
15	125.78	15808.6
16	128.84	16193.3
17	130.29	16376.0
18	131.33	16506.4
19	131.83	16569.6
20	133.40	16767.2
21	136.05	17100.0
22	137.91	17333.6
23	138.57	17416.2
24	142.01	17849.6
25	152.70	19192.4
26	169.01	21242.6

^{13}C NMR of compd **21** (125 MHz, DMSO-d_6).

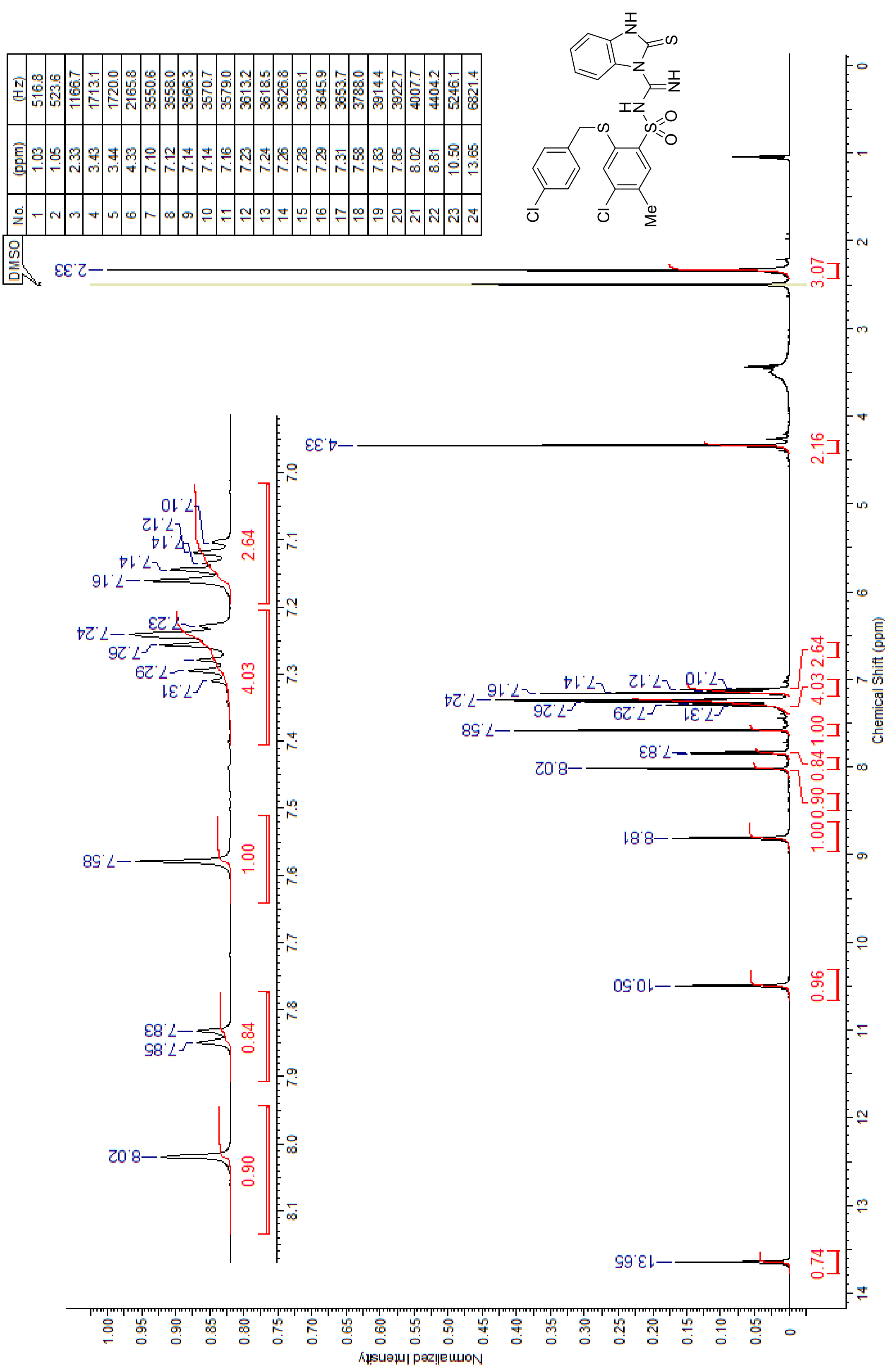

No.	(ppm)	(Hz)
1	1.03	516.8
2	1.05	523.6
3	2.33	1166.7
4	3.43	1713.1
5	3.44	1720.0
6	4.33	2165.8
7	7.10	3550.6
8	7.12	3558.0
9	7.14	3566.3
10	7.14	3570.7
11	7.16	3579.0
12	7.23	3613.2
13	7.24	3618.5
14	7.26	3626.8
15	7.28	3638.1
16	7.29	3645.9
17	7.31	3653.7
18	7.58	3788.0
19	7.83	3914.4
20	7.85	3922.7
21	8.02	4007.7
22	8.81	4404.2
23	10.50	5246.1
24	13.65	6821.4

^{1}H NMR of compd **22** (500 MHz, DMSO-d_6).

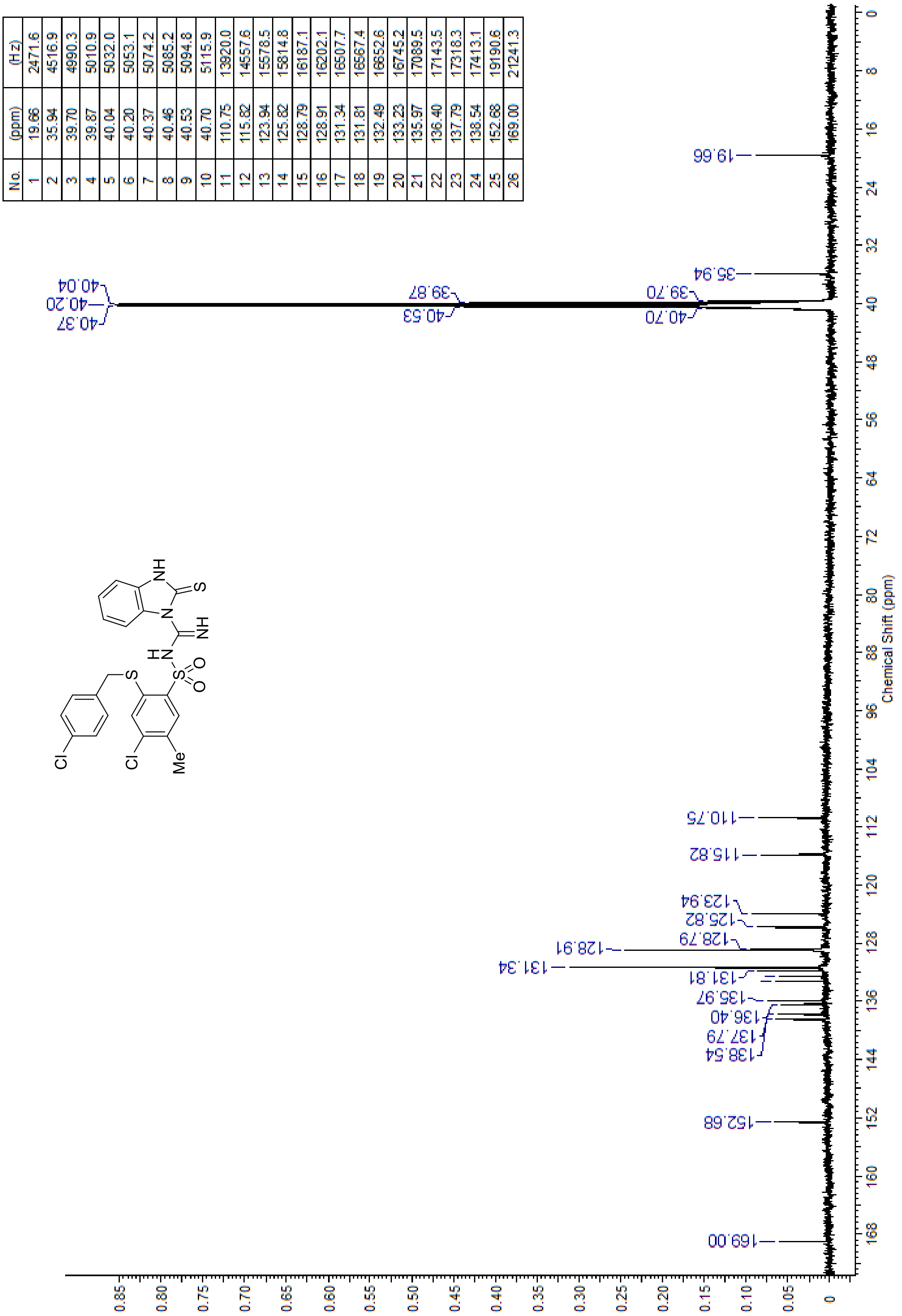

No.	(ppm)	(Hz)
1	19.66	2471.6
2	35.94	4516.9
3	39.70	4990.3
4	39.87	5010.9
5	40.04	5032.0
6	40.20	5053.1
7	40.37	5074.2
8	40.46	5085.2
9	40.53	5094.8
10	40.70	5115.9
11	110.75	13920.0
12	115.82	14557.6
13	123.94	15578.5
14	125.82	15814.8
15	128.79	16187.1
16	128.91	16202.1
17	131.34	16507.7
18	131.81	16567.4
19	132.49	16652.6
20	133.23	16745.2
21	135.97	17089.5
22	136.40	17143.5
23	137.79	17318.3
24	138.54	17413.1
25	152.68	19190.6
26	169.00	21241.3

^{13}C NMR of compd **22** (125 MHz, DMSO-d_6).

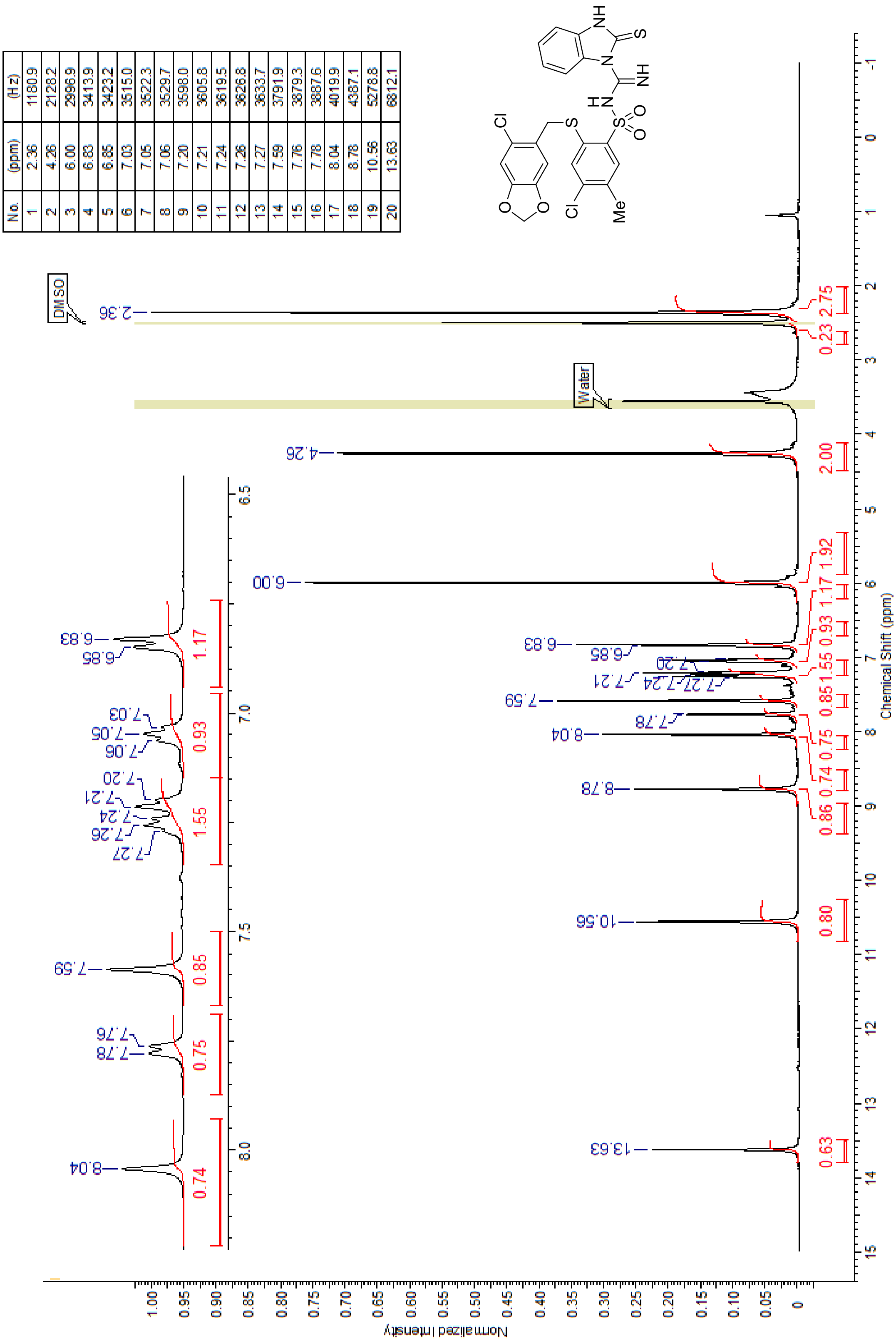

No.	(ppm)	(Hz)
1	2.36	1180.9
2	4.26	2128.2
3	6.00	2996.9
4	6.83	3413.9
5	6.85	3423.2
6	7.03	3515.0
7	7.05	3522.3
8	7.06	3529.7
9	7.20	3598.0
10	7.21	3605.8
11	7.24	3619.5
12	7.26	3626.8
13	7.27	3633.7
14	7.59	3791.9
15	7.76	3879.3
16	7.78	3887.6
17	8.04	4019.9
18	8.78	4387.1
19	10.56	5278.8
20	13.63	6812.1

^{1}H NMR of compd **23** (500 MHz, DMSO-d_6).

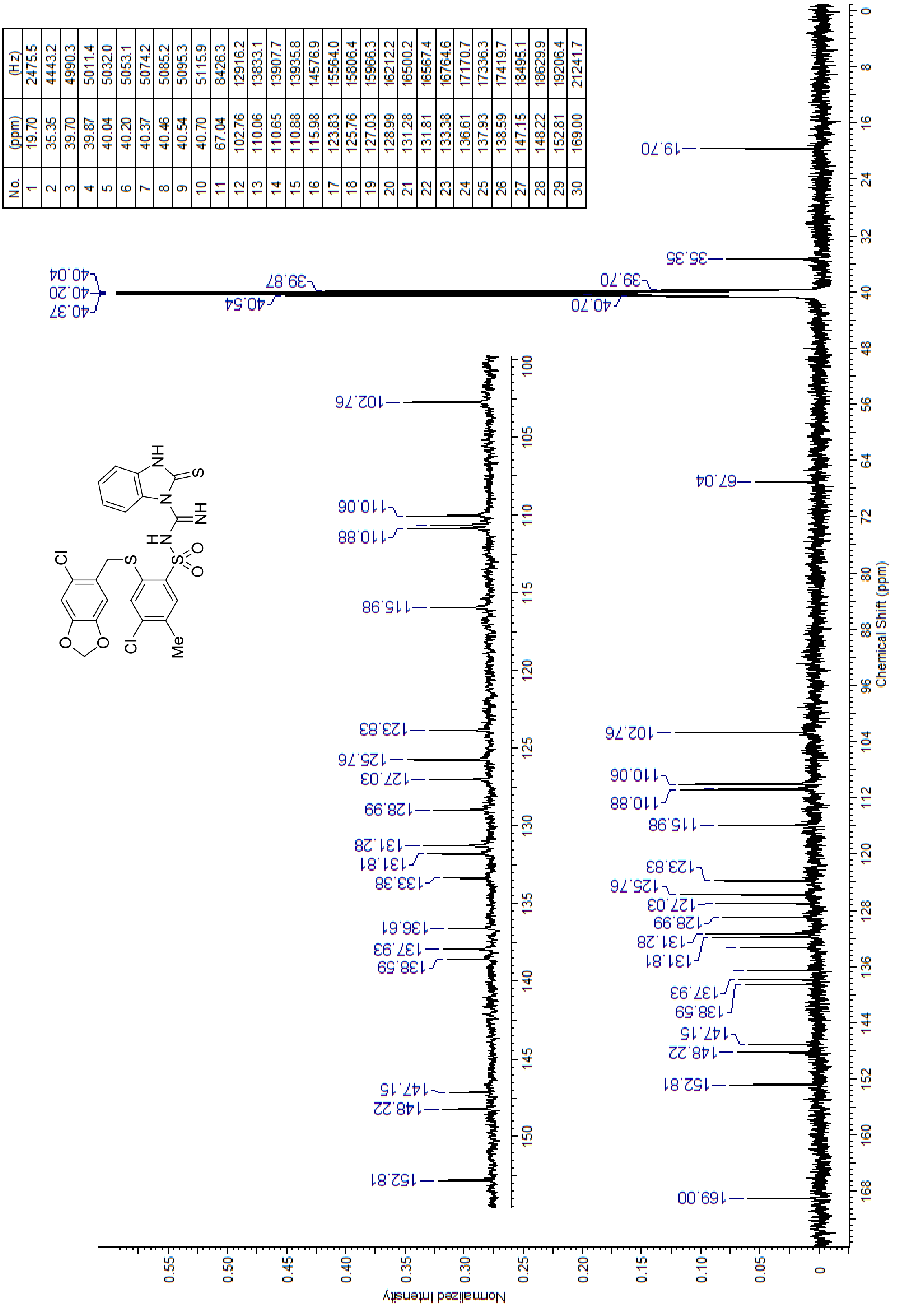

No.	(ppm)	(Hz)
1	19.70	2475.5
2	35.35	4443.2
3	39.70	4990.3
4	39.87	5011.4
5	40.04	5032.0
6	40.20	5053.1
7	40.37	5074.2
8	40.46	5085.2
9	40.54	5095.3
10	40.70	5115.9
11	67.04	8426.3
12	102.76	12916.2
13	110.06	13833.1
14	110.65	13907.7
15	110.88	13935.8
16	115.98	14576.9
17	123.83	15564.0
18	125.76	15806.4
19	127.03	15966.3
20	128.99	16212.2
21	131.28	16500.2
22	131.81	16567.4
23	133.38	16764.6
24	136.61	17170.7
25	137.93	17336.3
26	138.59	17419.7
27	147.15	18495.1
28	148.22	18629.9
29	152.81	19206.4
30	169.00	21241.7

^{13}C NMR of compd **23** (125 MHz, DMSO-*d*$_6$).
